# Long-term outcomes of rituximab, temozolomide and high-dose methotrexate without consolidation therapy for lymphoma involving the CNS

**DOI:** 10.2217/ijh-2017-0020

**Published:** 2018-01-26

**Authors:** Sarah J Nagle, Nirav N Shah, Alex Ganetsky, Daniel J Landsburg, Sunita D Nasta, Anthony Mato, Stephen J Schuster, Ran Reshef, Donald E Tsai, Jakub Svoboda

**Affiliations:** 1 Lymphoma Program, Abramson Cancer Center, University of Pennsylvania, Perelman Center for Advanced Medicine, 3400 Civic Center Blvd, 2 West Pavilion, Philadelphia, PA 19104, USA; 2 Medical College of Wisconsin, 9200 W. Wisconsin Ave, Milwaukee, WI 53226, USA; 3 Department of Pharmacy, Hospital of the University of Pennsylvania, 3400 Spruce Street, Philadelphia, PA 19104, USA; 4 Division of Hematology/Oncology & the Columbia Center for Translational Immunology, Department of Medicine, Columbia University Medical Center, New York, NY 10032, USA

**Keywords:** high-dose methotrexate, primary CNS lymphoma, secondary CNS lymphoma, temozolomide, treatment

## Abstract

**Aim:**

To describe the long-term outcomes of patients with lymphoma in the CNS treated with rituximab, temozolomide and high-dose methotrexate without consolidation therapy.

**Patients & methods:**

A retrospective cohort study of 46 consecutive patients with primary CNS lymphoma (PCNSL, 27 patients) or secondary CNS involvement of diffuse large B-cell lymphoma (DLBCL, 19 patients) who were treated with rituximab on day 1 in combination with high-dose methotrexate (days 1 and 15) and temozolomide (days 1–5) in 28-day cycles without further consolidation.

**Results:**

Median follow-up was 21.2 months. Patients received a median of five cycles (range 1–15). Median overall survival (OS) was 26 months and median progression-free survival was 8.6 months. At 3 years, 37% of patients were alive and without evidence of disease. The patients with PCNSL had a significantly higher response rates (ORR 81 vs 47%; p = 0.015) and longer median OS (55.3 vs 4.8 months; p < 0.01) than those with secondary CNS DLBCL. Toxicities were mild and manageable.

**Conclusion:**

The rituximab, temozolomide and methotrexate regimen is an effective therapy for patients with PCNSL without the toxicities typically associated with consolidation therapy.

Non-Hodgkin's lymphoma (NHL) can involve the CNS either as the sole area of disease or as a secondary spread of systemic disease. Primary CNS lymphoma (PCNSL) is a rare variant of NHL that involves the brain, leptomeninges, eyes or spinal cord without evidence of systemic disease [1]. It represents approximately 4% of newly diagnosed primary CNS tumors and 4–6% of all extranodal lymphomas [2,3]. Secondary involvement of the CNS by lymphoma presents heterogeneously with the most common manifestations being leptomeningeal disease and parenchymal brain involvement. Approximately 2–5% of patients with aggressive NHL develop involvement of the CNS at some point during their disease course [4–6].

Rapid control of CNS involvement by lymphoma is necessary to prevent significant neurologic morbidity. Standard immunochemotherapy regimens used in the treatment of systemic NHL are ineffective at controlling disease in the CNS since most of these agents do not penetrate through the blood–brain barrier [7]. There is no consensus on optimal therapy for PCNSL or secondary CNS involvement by diffuse large B-cell lymphoma (DLBCL), but National Comprehensive Cancer Network guidelines recommend a high-dose methotrexate-based regimen in eligible patients [8]. As the recommendations are broad, there is significant variation in clinical practice.

Intravenous methotrexate (MTX), given at sufficiently high doses to penetrate the CNS is the most active single agent against PCNSL and should be the backbone of induction therapy in most patients [9–11]. However, while high-dose methotrexate (HD-MTX)-based regimens are effective for induction therapy, the majority of patients with PCNSL who achieve a complete response (CR) will relapse [12]. Optimal consolidation therapy in this population has not been established.

Historically, whole-brain radiation therapy (WBRT) has been used for consolidation therapy in patients with PCNSL. However, while WBRT has been shown to improve progression-free survival (PFS), it does not improve overall survival (OS) compared with induction chemotherapy alone [13–15]. Additionally, there can be a high incidence of neurotoxicity and cognitive decline with consolidation radiation [16]. More recently, several groups have attempted to substitute WBRT consolidation with either high-dose chemotherapy with autologous stem cell transplant (HDT/ASCT) or nonmyeloablative chemotherapy (such as etoposide and cytarabine) [17–23]. These consolidation options can be associated with significant toxicity, especially in older patients with comorbidities.

Since 2009, we have used a prolonged course of the combination of rituximab, temozolomide and HD-MTX (RTM) without further consolidation to treat patients with PCNSL and secondary involvement of the CNS by DLBCL. Although it is clear that PCNSL and secondary involvement of the CNS by DLBCL are different entities in terms of biology, clinical history and outcome, we have chosen to present data for both in this manuscript as clinicians might be tempted to treat them the same based on their physical location. Here we report the results of our approach to these patient populations.

## Materials & methods

### Study design

We conducted a retrospective cohort study of consecutive patients with PCNSL or secondary CNS DLBCL who were treated with the RTM regimen at the Hospital of the University of Pennsylvania. The study was approved by the institutional review board of the University of Pennsylvania.

### Patients

Adult patients (18 years of age and older) were identified using a pharmacy database of patients who received HD-MTX, rituximab and temozolomide for PCNSL or secondary CNS DLBCL between 1 January 2009 and 31 December 2014. This would include almost all patients with CNS lymphoma treated during that time frame at our institution as RTM was our standard treatment regimen. To be included in the analysis, patients must have had documented lymphoma in their CNS (via biopsy or cerebrospinal fluid cytology). Patients were excluded if they received prior therapy for PCNSL or if they had prior CNS-directed therapy for active CNS involvement of DLBCL. Patients were also excluded if they had received radiation or HDT/ASCT as consolidation after frontline RTM for their lymphoma.

### Chemotherapy regimen

Patients were treated with rituximab 375 mg/m^2^ on day 1 in combination with HD-MTX 8 g/m^2^ (days 1 and 15) and temozolomide 150 mg/m^2^ (days 1–5) in 28-day cycles. HD-MTX was administered with leucovorin rescue. It was adjusted for creatinine clearance (Supplementary Table 1) and other toxicities (i.e., transaminitis, infection, poor performance status). Once a complete response (CR) was achieved, the day 15 MTX was omitted from subsequent cycles. Based on the protocol originally described in New Approaches to Brain Tumor Therapy (NABTT) 96–07, treatment was continued for 12 months unless there was disease progression or significant adverse events without further consolidation. Growth factor support was not mandatory and used at the discretion of the treating physician. Standard antimicrobial prophylaxis with fluconazole and antiviral antibiotics was used in all patients.

### Response & toxicity assessment

Initial response assessment was performed after one or two cycles of RTM with MRI of the brain or cerebrospinal fluid (CSF) cytology, where appropriate. Clinical response in the CNS was graded using the criteria from the International Workshop to Standardize Baseline Evaluation and Response Criteria for Primary CNS Lymphoma [24]. Toxicities were assessed and recorded using the Common Terminology Criteria for Adverse Events (CTCAE) Version 4 [25].

OS was defined as the time from initiation of therapy to last follow-up or death from any cause. PFS was calculated from initiation of therapy to disease progression or last follow-up.

### Statistical analysis

Descriptive and survival analyses using the Kaplan–Meier methodology were performed (STATA version 13; Stata Corp, TX, USA). A log-rank test was utilized to compare OS and PFS between patient groups. For the primary outcome of OS, we performed a univariate Cox proportional hazard analysis to evaluate the association of each variable on OS. All tests were two sided and a p-value of <0.05 was considered statistically significant.

## Results

### Patient characteristics

We identified 46 consecutive patients with PCNSL or secondary CNS DLBCL who received RTM at our institution between 1 January 2009 and 31 December 2014. 27 (59%) patients had PCNSL and 19 (41%) patients had secondary CNS DLBCL. The median age at diagnosis was 61 years (range: 21–85 years). The baseline characteristics at diagnosis are presented in Table 1. No patients received further consolidation chemotherapy or radiation therapy.

**Table 1.** **Baseline patient characteristics.**

**Characteristic**	**Number of patients**	**%**
Age at diagnosis:		

– >60 years	25	54

– ≤60 years	21	46

Gender:		

– Male	23	50

– Female	23	50

Disease:		

– PCNSL	27	59

– Secondary CNS DLBCL	19	41

Cycles of RTM received:		

– 1–2	16	35

– 3–5	9	20

– 6–9	7	15

– ≥10	14	30

Best response to therapy:		

– CR	26	56

– PR	11	24

– SD	5	11

– PD	4	9

CR: Complete response; DLBCL: Diffuse large B-cell lymphoma; PCNSL: Primary central nervous system lymphoma; PD: Progressive disease; PR: Partial response; RTM: Combination of rituximab, temozolomide and high-dose methotrexate; SD: Stable disease.

### Outcomes for the entire cohort

57% of patients achieved CR and 10.9% achieved partial response (PR) for an overall response rate (ORR) of 67.4%. The ORR in PCNSL was 81% versus 47.4% in secondary CNS DLBCL (p = 0.015). In patients achieving CR, the median time to best response was 2 months (two cycles). The patients received a median of five cycles (range 1–15) of RTM. Therapy was discontinued due to disease progression in 17 patients (seven [26%] PCNSL and ten  [53%] secondary CNS DLBCL). Therapy was discontinued in five (11%) additional patients due to acute kidney injury (AKI, three patients), pulmonary toxicity (one patient) and infection (one patient).

**Figure 1.**
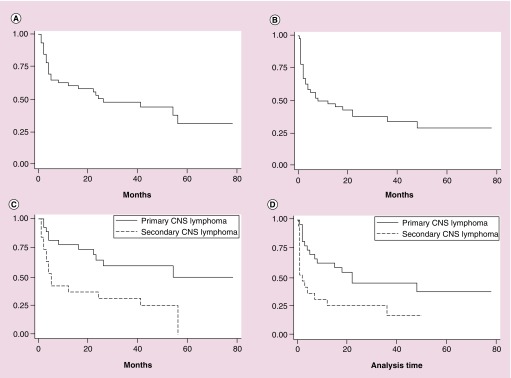
**Overall and progression-free survival.** **(A)** Overall survival of the entire cohort; **(B)** progression-free survival of the entire cohort; **(C)** overall survival by disease (primary versus secondary CNS lymphoma); and **(D)** progression-free survival by disease (primary versus secondary CNS lymphoma).

**Figure 2.**
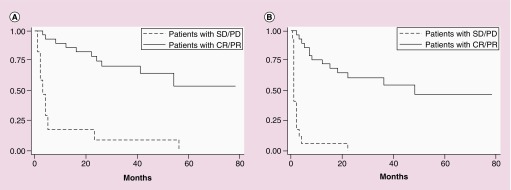
**Outcomes by response after two cycles of rituximab, temozolomide and high-dose methotrexate (RTM).** **(A)** Overall survival by response after two cycles of RTM. **(B)** Progression free survival by response after two cycles of RTM. CR: Complete response; PD: Progressive disease; PR: Partial response; SD: Stable disease.

For the entire cohort, the median OS was 26 months and median PFS was 8.6 months (Figure 1A & B). Compared with secondary CNS DLBCL, patients with PCNSL had a significantly longer median OS (55.3 vs 4.8 m; p < 0.01; see Figure 1C). PFS was also significantly longer for patients with primary versus secondary CNS DLBCL (22 vs 2 months; p = 0.02). The 4-year OS for the entire cohort was 44% (95% CI: 0.288–0.589). The 4-year PFS was 29% (95% CI: 0.153–0.45). Univariate cox proportional analysis demonstrated that sex and age did not impact OS. However, patients who were in a CR or PR after two cycles of RTM had a significantly improved OS compared with those who had stable disease (SD) or progressive disease (PD) at that time point (hazard ratio [HR]: 0.12; 95% CI: 0.05–0.29; p < 0.01; Figure 2 & Table 2).

**Table 2.** **Overall survival hazard ratios.**

**Variable**	**HR**	**p-value**	**95% CI**
Sex (female)	0.87	0.72	0.39–1.91

Not achieved CR	10.84	<0.001	4.21–27.91

Age >60	1.43	0.38	0.64–3.20

CR/PR after two cycles	0.12	<0.01	0.05–0.29

CR: Complete response; HR: Hazard ratio; PR: Partial response.

### Primary CNS lymphoma

27 (59%) patients had PCNSL and received RTM as their initial treatment. Patients received a median of seven cycles of RTM (range 2–15 cycles). Best response to therapy in this group was as follows: 19 (70%) patients had a CR, three (11%) patients had PR, three (11%) patients had SD and two (7%) patients had PD. The overall response rate in this patient cohort was 81%.

At 3 years, 16 of the 27 patients with PCNSL were alive (59%; 95% CI: 0.388–0.776). Out of the 16 patients with PCNSL, four patients had active disease (14%; 95%CI: 0.042–0.337); 12 patients had no evidence of active disease (44%; 95% CI: 0.255–0.647). The 4-year OS was 59% (95% CI: 0.373–0.761) and the 4-year PFS was 38% (95% CI: 0.178–0.582).

### CNS lymphoma secondary to DLBCL

19 (41%) patients had secondary CNS DLBCL and all had an isolated relapse in the CNS after prior systemic therapy with rituximab, cyclophosphamide, doxorubicin, vincristine and prednisone (R-CHOP). Patients received a median of two cycles of RTM (range 1–13 cycles). Best CNS response to therapy in this group was as follows: seven (37%) patients had CR, two (11%) patients had PR, one (5%) patient had SD and nine (47%) patients had PD. The ORR for patients with secondary CNS DLBCL was 47%. Ultimately, 15 of 19 (79%) patients had documented evidence of PD in the CNS. Two of these patients additionally had evidence of systemic progression.

Six of the 19 patients were alive at 3 years (31%; 95% CI: 0.1291–0.5225). One of the 19 patients was alive with active disease; the remainder were alive without active disease. The 4-year OS was 25% (95% CI: 0.086–0.462) and the 4-year PFS was 18% (95% CI: 0.038–0.396).

### Toxicity

Of the entire cohort, 33 (72%) patients developed an adverse event. This resulted in a dose decrease in 29 (63%) patients and termination of therapy in five (11%) patients. Toxicities included AKI, transaminitis, neutropenic sepsis, lung injury and mucositis. 20 patients (43%) developed AKI, with 95% of them requiring a dose decrease and three ultimately resulting in cessation of therapy. Transaminitis developed in seven (15%) patients, with 57% requiring a dose decrease and none resulting in termination of therapy. Four (9%) patients developed sepsis and therapy was discontinued in one (2%) patient as a result. One patient developed pulmonary toxicity and one patient developed mucositis. No patient deaths were considered to be treatment related. Adverse events resolved in all patients other than one patient with pulmonary toxicity who remained with persistent abnormalities on chest imaging but without clinical symptomatology. Complete toxicity data can be seen in Table 3. Given this was a retrospective analysis, effects of the chemotherapy on cognitive functions could not be assessed.

**Table 3.** **Toxicities.**

**Toxicity**	**Number of patients**	**%**
Acute kidney injury:		

– Grade 1	5	11

– Grade 2	12	26

– Grade 3	3	7

Transaminitis:		

– Grade 2	1	2

– Grade 3	6	13

Neutropenic sepsis:		

– Grade 4	4	9

Mucositis^†^	1	2

Pneumonitis^†^	1	2

^†^Unable to be graded.

## Discussion

We evaluated 46 consecutive patients treated at our institution between 2009 and 2014 with CNS involvement of their lymphoma who received RTM. 27 patients had PCNSL and 19 patients had secondary CNS lymphoma. The ORR for the entire cohort was 67%. At 3 years, 37% of patients (12 patients with PCNSL and five patients with secondary CNS lymphoma) were alive without evidence of disease. The 4-year OS for the entire cohort was 44%; it was 59% in the PCNSL cohort and 25% in the secondary CNS lymphoma cohort. The regimen was well tolerated.

Therapy for PCNSL often involves both methotrexate-based induction and further consolidation therapies. However, the original NABTT 96–07 study which established methotrexate monotherapy as the standard for PCNSL utilized 12 months of continuous methotrexate treatment with no consolidation. Since that time, clinicians have attempted to improve upon those results by adding other agents to methotrexate induction or incorporating a variety of consolidation therapies. The addition of rituximab to cytotoxic chemotherapy has been found to be safe and effective for PCNSL [26–28]. Studies have shown that consolidation with WBRT results in a median survival of 36–60 months [9,29,30]. However, recurrent or progressive disease is common and there is a substantial risk of late neurotoxicity particularly in patients older than 60 years. More recent data have suggested that HDT/ASCT is associated with high rates of remission. Several prospective Phase II trials and multicenter retrospective studies have reported high rates of long-term disease control [17,19,22,23,31,32]. However, this strategy is reserved for younger patients without comorbidities and generally these studies included only patients with PCNSL. Nonmyeloablative consolidation chemotherapy with cytarabine and etoposide is a feasible option in patients who achieve remission with induction therapy resulting in a median PFS of 4 years with a 4-year OS estimated at 65% [21]. However, this approach can be associated with significant toxicities including cytopenias, febrile neutropenia and death.

Optimal consolidation for older adults who achieve remission with induction chemotherapy has not been established. WBRT in patients older than 60 years is associated with the greatest risk of neurotoxicity including incontinence, gait instability and memory disturbances [33]. Additionally, this population often has a worse performance status compared with younger patients, which increases toxicity and limits their ability to tolerate HDT/ASCT and nonmyeloablative chemotherapy.

Given the small numbers of PCNSL patients and the difficulty in running clinical trials in this area, almost none of these newer therapeutic regimens have been tested in comparative trials. While consensus exists that induction consists of HD-MTX-based chemotherapy combinations, the optimal consolidation strategy and whether consolidation is even needed has not been established. Widely used consolidation strategies include WBRT, HDT/ASCT or nonmyeloablative chemotherapy, which add to the toxicity of high-dose methotrexate induction with unclear benefit. In this study, we present an alternative treatment strategy with the prolonged course of RTM, similar to that used in NABTT 96–07, without further consolidation [12].

Our results suggest that a prolonged course of RTM without consolidation therapy is a favorable treatment strategy for patients with PCNSL. This regimen is associated with improved ORR (81 vs 66%) and similar 4-year OS (59 vs 65%) as treatment regimens that use cytarabine and etoposide consolidation, but with less toxicity. This suggests that patients who respond initially to the combination of RTM may not need aggressive consolidation therapy such as etoposide and cytarabine, HDT/ASCT or WBRT. The RTM regimen may be a desirable option, particularly in patients who cannot tolerate HDT/ASCT or nonmyeloablative chemotherapy. Early response assessment can prognosticate patients. Patients who achieved CR within two cycles of therapy have an improved OS as compared with those who had only PR.

Logic would suggest that more is better and that by adding aggressive consolidation therapy to methotrexate-based induction, results would improve. However, in recent protocols that utilize consolidation steps, the number of high dose methotrexate cycles is reduced compared with that originally described in NABTT 96–07, which planned for year-long continuous methotrexate therapy. Whether reducing the number of methotrexate cycles in exchange for aggressive consolidation with WBRT, alternative chemotherapy or HDT/ASCT provides benefit and is worth the toxicity remains to be seen. Our study suggests there is substantial benefit in prolonged RTM induction without consolidation therapy in patients with PCNSL. This option may be especially useful in patients who would not otherwise tolerate consolidation therapy.

Secondary CNS DLBCL is associated with an extremely poor prognosis and a low likelihood of long-term survival [4]. In this study, the patients with relapsed DLBCL involving the CNS did not respond as well to RTM and despite CNS-directed therapy, all patients who had PD progressed in the CNS. This poor response in the CNS may represent a different biology and aggressive nature of the disease. Better treatment options need to be developed for this population.

The study has several limitations. It is a single institution retrospective cohort study and data were obtained via chart abstraction. Given it was not a prospective clinical trial, variations in dosing and scheduling occurred. Complete toxicity data may have been missing and other toxicities were not graded. Despite these potential limitations, we show that RTM without consolidation is a reasonable first-line treatment strategy for patients with PCNSL.

## Conclusion

In conclusion, we show that a prolonged course of RTM without consolidation is a favorable first-line treatment strategy for patients with PCNSL. It is associated with low rate of treatment discontinuation due to toxicity and its efficacy is within the range of previously described approaches that are frequently too toxic in older patients or those with comorbidities. Patients tolerated the therapy well, and those with toxicities to high-dose methotrexate were able to continue therapy with dose reductions and no long-term sequela. Early response assessment after two cycles of therapy was able to predict patients who would have improved OS. Future prospective studies are needed to validate these findings.

## Future perspective

Although the field of oncology is rapidly advancing over the recent years, CNS lymphoma has moved at a slower pace. Our paper brings up the issue of optimal initial therapy. There are currently multiple induction regimens available with multiple consolidation options afterward. However, unlike other areas in oncology where a standard of care has been established through the process of methodical comparative trials, the CNS lymphoma area is almost devoid of comparative trials. The cause of this of course is the rarity of the cancer type in combination with the lack of funding. As such, it is very unclear what optimal therapy in 2017 is for primary CNS lymphoma. Looking forward there is real need for large-scale collaborative comparative clinical to sort out optimal induction and consolidation steps. This issue is made even more urgent by the coming wave of newer novel agents, which have started to appear in the systemic lymphoma arena and will soon come into use for CNS lymphomas, further complicating treatment options. While currently confusion reigns and art may be as important as science for a clinician operating in this area, I believe the future is bright with a host of new options that once sorted out will ultimately lead to better outcomes for patients with CNS lymphoma.

**Summary points**

- Management of patients with primary CNS lymphoma (PCNSL) and those with secondary CNS involvement by diffuse large B-cell lymphoma (DLBCL) is challenging.
- This is a retrospective cohort study of 46 patients with PCNSL (27 patients) or secondary CNS DLBCL (19 patients) who were treated with rituximab on day 1 in combination with high-dose methotrexate (days 1 and 15) and temozolomide (days 1–5) in 28-day cycles without further consolidation.
- For the entire cohort, median overall survival (OS) was 41.8 months and median progression-free survival was 8.6 months.
- At 3 years, 33% of patients were alive and without evidence of disease.
- The patients with PCNSL had a significantly longer median OS than those with secondary CNS DLBCL.
- Patients who were in a complete or partial response after two cycles of rituximab, temozolomide and methotrexate had an improved OS compared with those who had stable or progressive disease.
- Acute toxicities included acute kidney injury, transaminitis, sepsis, mucositis and lung injury, but there were no long-term adverse effects of this regimen.
- The rituximab, temozolomide and methotrexate regimen without consolidation is an effective therapy for patients with PCNSL without the toxicities typically associated with consolidation therapy.
