# Supplementary material for: Long-term outcomes of rituximab, temozolomide and high-dose methotrexate without consolidation therapy for lymphoma involving the CNS
Source: Int J Hematol Oncol. 2018 Jan 26;6(4):113–21. doi: 10.2217/ijh-2017-0020 (PMC6171986; doi:10.2217/ijh-2017-0020)
Supplement: Supplementary file 1 [file ijh-06-113-s1.docx]

| **Supplementary Table 1. Methotrexate dose-adjustment based on renal function.** | |
| --- | --- |
| **Creatinine clearance (calculated by the Cockcroft–Gault equation)** | **Dose of methotrexate** |
| ≥ 100 ml/min | 8000 mg/m^2^ |
| 90 ml/min – 100 ml/min | 7200 mg/m^2^ |
| 80 ml/min – 90 ml/min | 6400 mg/m^2^ |
| 70 ml/min – 80 ml/min | 5600 mg/m^2^ |
| 60 ml/min – 70 ml/min | 4800 mg/m^2^ |
| 50 ml/min – 60 ml/min | 4000 mg/m^2^ |
| 40 ml/min – 50 ml/min | 3200 mg/m^2^ |
| < 40 ml/min | – |
